# Epidemiology of dog-mediated zoonotic diseases in Algeria: a One Health control approach

**DOI:** 10.1016/j.nmni.2019.01.001

**Published:** 2019-01-18

**Authors:** Moustafa Kardjadj, Meriem Hind Ben-Mahdi

**Affiliations:** 1) Ecole Supérieure en Sciences de l'Aliment et des Industries Agro-alimentaires, ESSAIA El-Harrach, Algeria; 2) Laboratoire de Recherche ‘Santé et Productions Animales’, Ecole Nationale Supérieure Vétérinaire (ENSV) d'Alger, El Harrach, Algiers, Algeria

**Keywords:** Algeria, dogs, One Health, zoonosis

## Abstract

Globally, human–dog interactions cause significant social, economic and human health costs. Public health problems linked with dogs include mainly zoonotic diseases. Recently the concept of a global and integrative approach to improve the health and well-being of people, animals and the environment has been strongly endorsed. This concept, often referred to as One Health, also reflects the collaboration in the field of surveillance and monitoring. Because humans and animals often suffer from the same pathogens and share the same environment, a cross-sector approach integrating human and animal disease surveillance information is required. The aims of the present study were to describe the incidence of dog-mediated zoonotic diseases in Algeria between 2010 and 2017, and to propose a One Health approach to control these diseases in Algeria. Rabies, leishmaniasis and echinococcosis are the major zoonoses in Algeria, with a reported average number of deaths per year, respectively, of 18, 7947 and 387. These zoonoses occur with the uncontrolled proliferation of household waste deposits, particularly in and around urban area which maintain the presence of stray dogs. The persistence of these diseases indicates the need for greater partnership and collaboration among multiple sectors, including medical doctors, veterinarians, ecologists, environmentalists and law-enforcement agents. Such partnerships permit the sharing of information, facilities and resources under a One Health approach; permit rapid communication among disciplines as well as interdisciplinary training/education opportunities and raising awareness among human population; and allow a combined effort towards disease surveillance/control, which will consequently improve the efficiency of the control programmes.

## Introduction

Dogs were domesticated by human approximately 14 000 years ago and become closely associated with human activities, including hunting, guarding and herding. This association was strengthened over time in many cultures, with dogs entering dwellings and spending time with humans, as well as acting as a social partner and companion [1]. However, when dogs are not provided with food and shelter by humans, they will roam. Semiroaming and free-roaming dog populations can create risks for public health as a result of zoonotic diseases [2]. Indeed, it is known that more than 60 zoonotic diseases transmitted to humans are dog mediated, including those of significant concern such as rabies, leishmaniasis and echinococcosis [2], [3].

Effective surveillance, prevention and control of zoonotic diseases pose a real challenge to the public health sector because of the wide variety of species involved and the often complex natural history of zoonotic agents [2]. The extensive patterns of global change; the increasing connections among humans, livestock, pets and wildlife; and transboundary trade and travel require a cross-sectoral, transnational concept of zoonosis control [4]. This concept, known as One Health, considers the notion that humans and animals often suffer from the same pathogens and share the same environment. A holistic cross-sector approach integrating human and animal disease surveillance information is therefore required [5].

Here we summarize the published literature on rabies, leishmaniasis and echinococcosis in Algeria. We also review data obtained from the annual epidemiology bulletin of the Algerian Institute of Public Health [6]. The objectives of this study were to describe the incidence of dog-mediated zoonotic diseases in Algeria and propose a One Health control approach.

## Epidemiology of major zoonotic diseases in Algeria

### Rabies

Data analysis from the annual epidemiology bulletin of the Algerian Institute of Public Health [6] from 2010 to 2017 (Table 1) revealed that humans die of rabies at a rate of 15 to 20 deaths per year, with an annual average of 18 deaths. Data analysis also revealed a significant recrudescence in postexposure prophylaxis (PEP) cases, from 97 321 in 2010 to 121 545 in 2017. The Algerian government covers the charges for PEP treatments, so the reported increase in PEP cases constitutes a huge burden for the government's budget [6]. Dog bites in Algeria have proven to be the source of PEP cases—an average of 80.43% cases reported during our study period [7].

**Table 1:** Annual incidence of rabies, leishmaniasis and echinococcosis in humans [6]

Year	No. of PEP cases (death)	No. of Leishmaniasis cases (VL)	No. of Echinococcosis cases
2010	97 321 (19)	11 734 (84)	309
2011	105 264 (18)	10 843 (53)	352
2012	109 811 (17)	9057 (48)	403
2013	114 652 (15)	6437 (52)	415
2014	118 456 (19)	5562 (30)	423
2015	120 532 (20)	7523 (36)	374
2016	120 952 (18)	7197 (41)	381
2017	121 545 (19)	7368 (32)	398
Average	112 154 (18)	7947 (47)	387

PEP, postexposure prophylaxis; VL, visceral leishmaniasis.

According to Algerian veterinary services, the number of vaccinated domestic carnivores increased from 640 805 in 2010 to 791 342 in 2015. Furthermore, an average of 22 163 carnivores were culled yearly between 2010 and 2015 [8]. Despite these continued efforts, the number of human and animal cases reported each year remains steady. Therefore, a paradigm shift is required to tackle human rabies in Algeria by focusing on immunizing the primary reservoir hosts—domestic dogs—rather than concentrating on PEP as the only efficient means to prevent human deaths from rabies [7].

### Leishmaniasis

Our analysis found a clear evidence of an increase in the *Leishmania* transmission rate in Algeria. The number of reported human cases from 2010 to 2017 ranged 5562 to 11 734, with an average of 7947 human cases per year (Table 1). Harrat et al. [9] reported that leishmaniasis is a reemerging disease in Algeria and seems to spread because of a combination of factors, including environmental changes as well as factors related to the immune status of the host and drug resistance. Furthermore, Mihoubi et al. [10] stated that leishmaniasis represents 35% of notifiable diseases in Algeria, leading to its first rank among parasitic diseases in Algeria. Dogs are one of the main reservoirs of the parasite, and *Phlebotomus* insects are the vectors. Adel et al. [11] reported a prevalence estimate of dog leishmaniasis of 11% to 38% in six localities along a west–east transect in the Algerian littoral zone.

Human visceral leishmaniasis (VL) caused by *Leishmania infantum* (MON-1 and MON-24), conveyed by *Phlebotomus perniciosus* with dogs as the reservoir, is the most severe form of leishmaniasis [10]. It has been recorded in the north of Algeria in a humid bioclimatic area, but it is spreading throughout the country [12]. Mortality is observed in children from 0 to 4 years old, in whom the incidence is 2.72 cases per 100 000 inhabitants [10]. Moreover, data obtained from the Algerian Institute of Public Health [6] from 2010 to 2017 showed an average of 47 cases (range, 30–84 cases) of VL per year.

Cutaneous leishmaniasis (CL), however, manifests as four clinical forms in Algeria, with the reservoir and vector varying from place to place [13]. The first form, zoonotic CL, caused by *Leishmania major,* is identified in semiarid and arid to Saharan regions; it is transmitted mainly by *Phlebotomus papatasi,* with the *Gerbillidae* as the main reservoir [9]. The second form, sporadic localized CL caused by *Leishmania infantum,* is inoculated by *Phlebotomus perfiliewi;* the geographic distribution and reservoir are similar to the first form [13]. The third type of CL, that due to *Leishmania killicki,* has been identified in Ghardaïa, southern Algeria; the reservoir is mainly the rodent *Massoutier amzabi,* and the vector is *Phlebotomus sergenti* [14]. The fourth form is caused by *Leishmania tropica* and was noticed in urban areas [15]. The burden of CL has been rapidly increasing during the last decade, with an enzootic mode. The number of cases from 2010 to 2017 ranged between 5532 and 11 650, with an average of 7947 cases per year. CL, also called Biskra boil, represents a real public health problem in Algeria, which has the second largest focus in the world after Afghanistan [6].

### Echinococcosis

Cystic echinococcosis (CE) is a widespread zoonosis caused by *Echinococcus granulosis.* The adult worm lives in the small intestine of carnivores (mostly dogs). The intermediate larval stage develops in the internal organs of many mammal species (including humans), which acquire the infection through accidental ingestion of tapeworm eggs [16]. CE is distributed worldwide and is prevalent mostly in Mediterranean countries. It is one of the major parasitic diseases in the Middle East and North Africa. Although both cystic and alveolar echinococcosis have been reported in these areas, CE is the most prevalent form [17], [18].

Algerian Institute of Public Health [6] data show the enzootic trend of CE incidence in Algeria: from 2010 to 2017 the annual number of human cases ranged between 309 and 423, with an average of 387 cases per year (Table 1). The persistence of the infection may be mainly explained by stray dogs eating offal discarded from animals slaughtered for human consumption, which contributes to maintain the life cycle of *Echinococcus granulosis* [6]. In Algeria, CE is a serious economic and public health problem because of the huge medical fees related to surgical treatment and livestock losses. The common sheep–dog cycle continues to be the major source of human contamination [17].

## Proposition of a One Health control approach

The One Health concept calls for a paradigm shift in developing, implementing and sustaining health policies that implement collective and coordinated actions among human, animal and environment sectors [5]. The One Health concept helps us understand the interactions among animals, humans and the environment and how these interactions affect the occurrence of infectious diseases. It aims to promote interdisciplinary collaborations among wildlife biologists, behavioural scientists, ecologists, agriculturalists, veterinarians, physicians, virologists, biomedical engineers and epidemiologists, among others, to attain optimal health for humans, animals and the environment [4].

According to the Algerian Institute of Public Health [6], the major zoonotic diseases in Algeria (rabies, leishmaniasis and echinococcosis) are dog mediated. The occurrence of these diseases is helped along by the presence and uncontrolled proliferation of household waste deposits coupled with semiroaming and free-roaming dogs [2], [3]. The proportion of this population varies considerably from one country to another. In Algeria, the size of the semiroaming and free-roaming dog population is unknown, and there is no official vaccination programme for this category of animal [8]. Furthermore, understanding the demographic characteristics of dog populations may greatly affect the transmission and maintenance of pathogenic agents. Such knowledge can influence logistics, such as the quantity of vaccines required and the frequency of vaccination campaigns [7].

Control and eradication of zoonotic diseases are desired by many countries where these diseases are endemic. However, this result is difficult and expensive. The following must be taken into account: specific climatic, geographical and socioeconomic profiles; animal ownership; existence of technical resources and personnel; local disease prevalence; and strict commitment to vaccination programmes. Developing countries thus have major difficulties in organizing and achieving success in the control and eradication programmes of this disease [19].

In order to develop an effective One Health implementation plan for strengthening capacity at the Algerian national level among multiple sectors to control these zoonotic diseases, we need to adopt an intersectoral collaboration that includes medical doctors, veterinarians, ecologists, environmentalists and law-enforcement agents that can be translated to

• Sharing information, facilities and resources under a One Health approach.
• Communicating rapidly among disciplines, providing interdisciplinary training/education and raising awareness among the human population.
• Combining efforts towards disease surveillance/control and continuous sustained interventions.

Collaboration among the departments of health, veterinary services and the environmental agency is important for the control of zoonoses in Algeria and thereby the elimination of transmission to humans. Periodical joint meetings will be of mutual benefit for both services and the general public. Open communication and integration of surveillance and monitoring systems for both humans and animals will be mutually beneficial. A detailed epidemiologic investigation focusing on host, agent and environmental factors needs to be performed throughout the country in order to identify the risk factors associated with transmission and maintenance of these dog-mediated zoonoses. It is critical that all sectors cooperate in these efforts to

• Ensure better understanding and management of household waste.
• Describe the demographic characteristics of dog populations and stray animals.
• Ensure there is at least one animal shelter per district.
• Enforce the mandatory nature of rabies vaccination and pest-control collars for carnivores.
• Strengthen anti-*Phlebotomus* control operations to fight leishmaniasis.
• Destroy viscera by rendering or deep burial.
• Encourage citizens to be active players in prevention through health education.

Awareness programmes and public outreach remain essential elements for the success of campaigns and eradication of these dog-mediated zoonotic diseases.

## Conflict of interest

None declared.
